# Traumatic Brain Injury and Acute Kidney Injury—Outcomes and Associated Risk Factors

**DOI:** 10.3390/jcm11237216

**Published:** 2022-12-05

**Authors:** Jesús Abelardo Barea-Mendoza, Mario Chico-Fernández, Manuel Quintana-Díaz, Lluís Serviá-Goixart, Ana Fernández-Cuervo, María Bringas-Bollada, María Ángeles Ballesteros-Sanz, Íker García-Sáez, Jon Pérez-Bárcena, Juan Antonio Llompart-Pou

**Affiliations:** 1 UCI de Trauma y Emergencias, Servicio de Medicina Intensiva, Hospital Universitario 12 de Octubre, 28041 Madrid, Spain; 2 Servicio de Medicina Intensiva, Hospital Universitario La Paz, 28029 Madrid, Spain; 3 Servei de Medicina Intensiva, Hospital Universitari Arnau de Vilanova, Universitat de Lleida, IRBLleida, 25198 Lleida, Spain; 4 Servicio de Medicina Intensiva, Hospital Universitario Puerta del Mar, 11009 Cádiz, Spain; 5 Servicio de Medicina Intensiva, Hospital Clínico Universitario San Carlos, 28040 Madrid, Spain; 6 Servicio de Medicina Intensiva, Hospital Universitario Marqués de Valdecilla, 39008 Santander, Spain; 7 Servicio de Medicina Intensiva, Hospital Universitario de Donostia, 20014 Donostia, Spain; 8 Servei de Medicina Intensiva, Hospital Universitari Son Espases, Institut d’Investigació Sanitària Illes Balears (IdISBa), 07120 Palma, Spain

**Keywords:** traumatic brain injury, severe trauma, acute kidney injury, intensive care, mortality, RETRAUCI

## Abstract

Our objective was to analyze the contribution of acute kidney injury (AKI) to the mortality of isolated TBI patients and its associated risk factors. Observational, prospective and multicenter registry (RETRAUCI) methods were used, from March 2015 to December 2019. Isolated TBI was defined as abbreviated injury scale (AIS) ≥ 3 head with no additional score ≥ 3. A comparison of groups was conducted using the Wilcoxon test, chi-square test or Fisher’s exact test, as appropriate. A multiple logistic regression analysis was conducted to analyze associated risk factors in the development of AKI. For the result, overall, 2964 (30.2%) had AIS head ≥ 3 with no other area with AIS ≥ 3. The mean age was 54.7 (SD 19.5) years, 76% were men, and the ground-level falls was 49.1%. The mean ISS was 18.4 (SD 8). The in-hospital mortality was 22.2%. Up to 310 patients (10.6%) developed AKI, which was associated with increased mortality (39% vs. 17%, adjusted OR 2.2). Associated risk factors (odds ratio (OR) (95% confidence interval)) were age (OR 1.02 (1.01–1.02)), hemodynamic instability (OR 2.87 to OR 5.83 (1.79–13.1)), rhabdomyolysis (OR 2.94 (1.69–5.11)), trauma-associated coagulopathy (OR 1.67 (1.05–2.66)) and transfusion of packed red-blood-cell concentrates (OR 1.76 (1.12–2.76)). In conclusion, AKI occurred in 10.6% of isolated TBI patients and was associated with increased mortality.

## 1. Introduction

Medical complications are common in traumatic brain injury (TBI) patients and potentially influence outcomes [1]. Specifically, acute kidney injury (AKI), which is common in trauma patients in our environment [2,3], can appear in 8–23% of TBI patients through mediating different complex mechanisms [4,5]. In summary, two main pathophysiological processes might lead to AKI [5]. The first is the neuroendocrine pathway, which includes the central nervous autonomous system and the endocrine system, and the second includes the inflammatory and immune pathways [5,6].

Specifically, trauma might be associated with intravascular volume depletion, raised intra-abdominal pressure, rhabdomyolysis or sepsis, which are known to be associated with the development of AKI [6]. Iatrogenic factors, including exposure to radiocontrast agents, nephrotoxic antibiotics and anti-inflammatory agents [6], must also be considered in brain-injured patients. The treatment of intracranial hypertension may also contribute to the development of AKI [7].

AKI was found to be an early phenomenon that negatively affected mortality and six-month neurological outcomes in TBI patients [8]. However, in this study, patients with associated major extracranial injuries that could play a role in the incidence of AKI were included. In addition, Luu et al. reported an incidence of severe AKI of 2.1% in patients with isolated severe TBI [9]. In their study, TBI patients who developed severe AKI had a higher in-hospital mortality and higher healthcare resource utilization [9]. However, diagnosis of severe AKI was based in administrative data and coding diagnosis, and therefore, therapies used to treat TBI and milder forms of AKI were not analyzed [9].

Our objective was to analyze the contribution of acute kidney injury (AKI) to the mortality of isolated TBI patients and its associated risk factors in a large multicenter sample of TBI patients included in the Spanish trauma ICU registry (RETRAUCI).

## 2. Materials and Methods

The observational, prospective and multicenter registry included 52 ICUs in Spain. It works in a web-based electronic database (retrauci.org) and is endorsed by the Neurointensive Care and Trauma Working Group of the Spanish Society of Intensive Care Medicine (SEMICYUC). The study was conducted in accordance with the Declaration of Helsinki, and the registry was approved by the Ethics Committee of Hospital Universitario 12 de Octubre, Madrid (12/209). Due to the retrospective analysis of deidentified data, informed consent was not obtained.

All patients admitted to the participating ICUs from March 2015 to December 2019 who presented isolated significant TBI, defined as an abbreviated injury scale (AIS) ≥ 3 in the head area, were included. Patients with AIS head < 3 or patients with AIS ≥ 3 in any other anatomical area were not included in this study. Patients were managed according to the advanced trauma life support (ATLS) principles. Data on epidemiology, acute management in the prehospital and in-hospital stages, type and severity of injury, resource utilization, complications and outcomes were recorded. Patients were followed up with until hospital discharge.

In this population, we analyzed the impact of AKI at any stage during hospitalization, evaluated with the risk, injury, failure, loss of kidney function and end-stage kidney disease (RIFLE) definition [10], which evaluates glomerular filtration rate (GFR) and urinary output (UO) criteria and includes the following categories [10]:

Risk: serum creatinine × 1.5 or GFR decrease by 25%. UO < 0.5 mL/kg/h × 6 h.

Injury: serum creatinine × 2 or GFR decrease > 50%. UO < 0.5 mL/kg/h × 12 h.

Failure: serum creatinine × 3, or GFR decrease by 75% or serum creatinine ≥ 4 mg/dL with an acute rise > 0.5 mg/dL. UO < 0.3 mL/kg/h × 24 h, or anuria × 12 h.

Loss: persistent acute renal failure = complete loss of kidney function > 4 weeks.

End-stage kidney disease: end-stage kidney disease > 3 months.

Where unknown, an estimation of the baseline creatinine values was calculated by using the modification of diet in renal disease (MDRD) formula (GFR was accepted to be 75 mL/min/1.73 m^2^).

The following lists other definitions [2].

− Hemodynamic condition was considered as follows:

Stable: systolic blood pressure > 90 mmHg during the first medical attention after trauma.

Unstable, responding to volume replacement: systolic blood pressure < 90 mmHg requiring volume replacement for normalization.

Shock: systolic blood pressure < 90 mmHg requiring volume replacement, blood products and vasoactive support for normalization.

Refractory shock: hypotension refractory to volume replacement, blood products or vasoactive support and activation of the massive bleeding protocol.

− Multiorgan failure was defined as using the sequential-related organ failure assessment (SOFA) as the alteration of two or more organs with a maximum score ≥ 3 [11]. Early MOF was that occurring in the first 72 h after trauma. Late MOF occurred beyond day 3 post-trauma [11].
− Rhabdomyolysis: laboratory test determination of creatine kinase > 5000 U/L [2].
− Trauma-associated coagulopathy: prolongation of the prothrombin and activated partial thromboplastin times over 1.5 times the control values, or fibrinogen < 150 mg/dL or thrombocytopenia (<100,000/µL) in the first 24 h.

### Statistical Analysis

Quantitative variables are shown as mean ± standard deviation (SD), median (interquartile range) and qualitative variables as percentage. Groups with quantitative variables were compared using Wilcoxon test and groups with categorical variables using the chi-square test or Fisher’s exact test, as appropriate. The associated risk factors of developing AKI were analyzed by using multiple logistic regression analysis. The variables entered in the model were those associated with AKI in the univariate analysis (with *p*-value < 0.10). We analyzed the contribution of AKI to crude and adjusted mortality by using logistic regression analysis. A *p* < 0.05 was considered statistically significant. We reported all results as stated in the RECORD statement [12]. The statistical analysis was performed using STATA 15 (StataCorp., TX, USA, 2017).

## 3. Results

During the study period, 9790 trauma patients were admitted. Among them, 2964 (30.2%) had AIS head ≥ 3 with no other area with AIS ≥ 3 and constituted the study cohort. The mean age was 54.7 (SD 19.5) years, 76% were men, and the main mechanism of injury was ground-level falls (49.1%). Only 2.6% of the cases had a penetrating trauma. Overall, 22% were receiving chronic treatment with antiplatelets or anticoagulants. The median Glasgow coma score was 11 (interquartile range 6–14). Up to 26.2% of the patients received prehospital intubation. The mean injury severity score (ISS) was 18.4 (SD 8). The mean new injury severity score (NISS) was 27.04 (SD 13.89). In 28.2% of the cases, an early (<24 h) neurosurgical procedure was necessary. Invasive neuromonitoring was used in 30.65% of the patients. Up to 62.5% received mechanical ventilation during their ICU stay. In this group, mechanical ventilation was used for 8.24 (SD 9.23) days. Tracheostomy was necessary in 14.18% of the whole sample. The length of patients’ ICU stays averaged 9.61 (SD 14.22) days. In-hospital mortality was 22.2%. The main reason for mortality was intracranial hypertension (65.99% of the deceased).

Overall, 310 of the 2964 patients (10.6%) developed AKI, distributed by the categories of risk (166 patients, 5.68%), injury (80 patients, 2.74%) and failure (44 patients, 1.50%). Up to 20 additional TBI patients were classified in the loss or end-stage kidney disease categories.

Patients who developed AKI were older (mean age 61.85 vs. 53.87 years, *p* < 0.001), had a higher severity of injury (mean ISS 20.85 vs. 18.10, *p* < 0.001 and mean NISS 29.59 vs. 26.73, *p* < 0.001), required more days of mechanical ventilation (10.7 vs. 7.8 days, *p* < 0.001) and had a longer ICU stay (13.1 vs. 9.2 days, *p* < 0.001). Prehospital intubation (33.66 vs. 25.26%, *p* < 0.001), hemodynamic instability (50.32 vs. 21.17%, *p* < 0.001), transfusion of packed red-blood-cell concentrates in the initial 24 h after injury (19.35 vs. 8.19%, *p* < 0.001) and transfusion of fresh frozen plasma in the initial 24 h (11.27% vs. 6.94%, *p* = 0.023) were more frequent in the AKI group. Once brain-injury-related variables have been factored in, the severity of brain injury for AKI patients had increased, as stated by the higher number of patients with Glasgow coma scale <9, the higher number of patients with pupillary abnormalities, the higher use of invasive intracranial pressure (ICP) neuromonitoring and the higher scores in the AIS head (Table 1).

No differences were found in sex, blood pressure, cardiac rate, shock index or respiratory rate at the initial medical attention. Ethanol poisoning was more frequent in non-AKI patients (24.32 vs. 15.85%, *p* < 0.001).

Isolated TBI patients with AKI were more likely to present different complications (Table 2).

During patients’ respective ICU stays, only 15 of them with isolated TBI received renal replacement therapies (RRT) because of AKI (0.5% of the whole sample of TBI and 4.8% of those who developed AKI).

**Figure 1:**
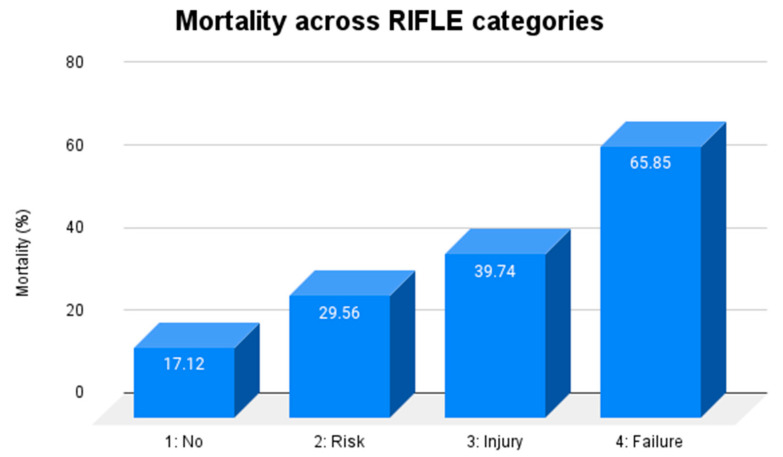
Mortality rate according to the development of AKI in TBI patients. RIFLE: Risk, Injury, Failure, Loss of kidney function and End-stage kidney disease.

AKI was associated with increased mortality (39% vs. 17%, *p* < 0.001), even after adjusting for confounding factors such as age and AIS head (OR 2.22 (1.64–2.99). Mortality increased with higher stages of the RIFLE classification, ranging from 17.1% in TBI patients without AKI to 65.8% in the failure category (Figure 1).

**Figure 2:**
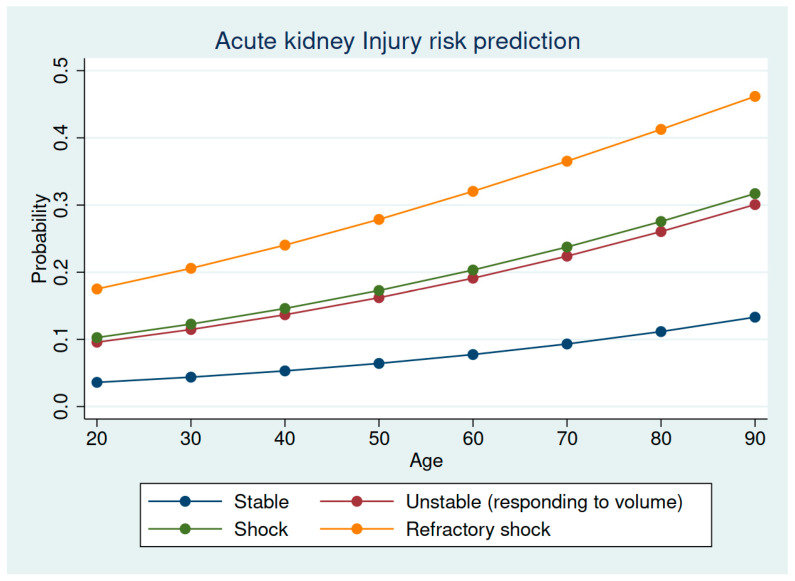
Risk of developing AKI according to age and the hemodynamic response.

The risk factors associated with the development of AKI in isolated significant TBI patients are shown in Table 3. The probability of developing AKI was strongly associated with age and the initial hemodynamic response (Figure 2).

## 4. Discussion

The main findings of our study were that AKI was found in one in 10 patients with isolated significant TBI, similar to what was previously described in smaller samples of TBI patients. AKI was strongly associated with mortality. The logistic regression analysis identified the following clinical factors as being associated with AKI in isolated TBI: age, hemodynamic instability, rhabdomyolysis, trauma-associated coagulopathy and the early need for packed red-blood-cell concentrates.

The pathophysiology of AKI in the neurocritically ill patients is complex and multifactorial [4,5]. The severity of a brain injury constitutes a major determinant of developing AKI, and in turn, AKI may aggravate brain injury, leading to a vicious circle [13]. In summary, the neuroendocrine pathway, including the central nervous autonomous system and the endocrine system, and the inflammatory and immune responses play a major role in its development [5,6]. Patient-, trauma-, iatrogenic- or treatment-related factors may contribute [5,6,7]. Indeed, the Collaborative European NeuroTrauma Effectiveness Research in Traumatic Brain Injury (CENTER-TBI) showed that an AKI incidence of 12% of cases in a sample of 1262 TBI patients was associated with patients’ previous conditions (renal history and insulin-dependent diabetes), trauma (pupillary reactivity) and treatments received (osmotic therapy and natremia ≥ 150 mmol/L in the initial 3 days after injury) [8]. More recently, Luu et al., in the largest sample of severe TBI patients, found that 2.1% of patients had severe AKI (acute kidney injury network stage 3 or higher) [9]. We observed an intermediate incidence of AKI (10.6%) that was related to patient factors (age) and the physiological response to trauma (hemodynamic instability, trauma-associated coagulopathy and the early need for packed red-blood-cell concentrates) and trauma (rhabdomyolysis), even after excluding patients with major extracranial injuries. Our population was similar to that in the study of Luu et al. [9] in age and inclusion criteria. If we were to analyze only the incidence of AKI in the failure category, we would obtain a similar incidence, of 1.5%. However, we must keep in mind that Luu et al. [9] and our study used different definitions and therefore could not reflect the same subset of TBI patients.

Some points need to be addressed to put our results into context. First, our incidence was lower in isolated TBI patients than in the whole sample of trauma patients, including those with major extracranial injuries, despite the fact that some associated risk factors were the same [2]. Second, compared with the CENTER-TBI study [8], our patients were older (and older age is a well-known risk factor for developing AKI [3]). In injured patients aged ≥75 years, AKI was associated with the severity of injury and shock rather than comorbidities and with increased risk of death [14]. On the other hand, patients in the CENTER-TBI database were more severely injured, as noted by their higher ISS, and had major extracranial injuries in 62% of the patients, which constitutes an exclusion criterion in our sample of isolated TBI patients, as done by Luu et al. [9]. Even with this exclusion criterion, clinical factors reflecting a shock state, such as hemodynamic instability, trauma-associated coagulopathy, rhabdomyolysis and the early need for packed red-blood-cell concentrates, were found to be associated with developing AKI in isolated TBI patients [15,16], which are similar to our results, in that we observed a strong relationship of AKI with age and hemodynamic response (see Figure 2). Of note, despite AKI patients’ having had increased severity of brain injury (higher percentage of patients with Glasgow coma scale < 9, pupillary abnormalities, with ICP monitoring and higher scores in the AIS head) these specific brain-injury-related variables were not significantly associated with developing AKI according to the multivariate analysis. More intriguing results showed the protective effect of the early transfusion of fresh frozen plasma with relation to developing AKI. Early transfusion of plasma has been associated with improved selective outcomes in TBI patients [17,18]. Although our study is not designed for this purpose, we speculate that early plasma transfusion could be associated with an improved hemodynamic condition or with a limited progression of intracranial injuries and, therefore, a reduced incidence of AKI, given that it was not associated with AKI in our prior study of a general trauma ICU sample [2]. Taking together and considering the high impact of AKI in mortality and the associated risk factors found in our study, we recommend administering a balanced resuscitation in the case that blood products are necessary, because age, rhabdomyolysis and hemodynamic instability are nonmodifiable factors.

We observed a low incidence of renal replacement in our patients, as found in previous studies [8,19]. The need of RRT in acute brain injury constitutes a challenge in patients with acute brain injury, because of the potential derangements of the technique on ICP dynamics [20,21]. The low incidence found in our series precludes further analysis of this subgroup.

Strengths of our study include a large sample of TBI patients with a classification of AKI according to the RIFLE criteria. We believe that it delineates the epidemiology of AKI in isolated TBI patients, supporting the usefulness of trauma registries in the management and benchmarking of severe trauma patients [22]. However, we must also keep in mind our limitations: baseline creatinine was not available for all patients, so the RIFLE criteria may have misclassified our TBI patients. Second, the definition currently accepted by the KDIGO guidelines was not used, because it was not available at the time the registry was designed [23]. Third, unless patients are initially managed following the ATLS principles, we cannot rule out variability among centers that could potentially affect the management of patients and the AKI incidence. Fourth, the current design of the trauma registry does not account for comorbidities, which have been well-known risk factors for posttraumatic AKI in previous studies [8,9]. Because of the same design issues, temporal trends of AKI cannot be analyzed.

## 5. Conclusions

In our study, including a large sample of isolated TBI patients admitted to the ICU, AKI was detected in 10.6% and was associated with increased crude and adjusted mortality. Age, hemodynamic instability, rhabdomyolysis, trauma-associated coagulopathy and the early need for the transfusion of red-blood-cell concentrates were associated risk factors of developing AKI.

## Figures and Tables

**Table 1:** The distribution of the severity of brain injury evaluated by the initial Glasgow coma scale, the pupillary response, the abbreviated injury scale in the anatomical head area and the need for invasive ICP neuromonitoring according to the presence of AKI.

Variable	Non-AKI	AKI	*p*-Value
GCS			<0.01
GCS 14–15	35.95%	24.32%	
GCS 9–13	26.91%	27.40%	
GCS ≤ 8	37.14%	48.29%	
Pupillary status			<0.01
Both reactive	81.85%	72.4%	
Unilateral mydriasis	11.91%	15.58%	
Bilateral mydriasis	6.24%	12.01%	
AIS head			<0.001
AIS head 3	36.27%	22.58%	
AIS head 4	31.48%	30.65%	
AIS head 5	31.71%	45.48%	
AIS head 6	0.54%	1.29%	
Invasive ICP neuromonitoring	29.74%	35.69%	0.035

AKI: acute kidney injury; GCS: Glasgow coma scale; AIS: abbreviated injury scale; ICP: intracranial pressure.

**Table 2:** The distribution of complications in isolated TBI patients distributed by the existence of AKI.

Variable	Non-AKI	AKI	*p*-Value
Rhabdomyolysis	3.14%	9.68%	<0.01
Trauma-associated coagulopathy	7.65%	17.74%	<0.01
PO_2_/FiO_2_ < 300	18.22%	43.04%	<0.01
Nosocomial infection	25.16%	40.33%	<0.01
Multiorgan failure	4.02%	26.21%	<0.01

AKI: acute kidney injury.

**Table 3:** The results of the logistic regression analysis aiming to identify risk factors associated with AKI.

	OR (95% CI)	*p*-Value
Age (per year)	1.02 (1.01–1.02)	<0.001
Rhabdomyolysis	2.94 (1.69–5.11)	<0.001
Transfusion of packed red-blood cells < 24 h)	1.76 (1.12–2.76)	0.027
Transfusion fresh frozen plasma (<24 h)	0.76 (0.61–0.94)	0.012
Trauma-associated coagulopathy	1.67 (1.05–2.66)	0.029
Hemodynamic instability		
Volume-responding	2.87 (1.79–4.60)	<0.001
Shock	3.10 (2.16–4.46)	<0.001
Refractory shock	5.83 (2.60–13.1)	<0.001

Values in parentheses are 95% confidence intervals. Area under receiver operating characteristic curve (AUROC 0.72 (0.68–0.76)), Hosmer–Lemeshow (HL) χ^2^ = 8.30, *p* = 0.40.

## Data Availability

Data are available from the corresponding author upon reasonable request.
